# Letter to the Editor regarding the article by Borkens Y, Endruscheit U, Lübbers CW. Homeopathy—A lively relic of the prescientific era. Wien Klin Wochenschr 2023:1–8

**DOI:** 10.1007/s00508-023-02233-0

**Published:** 2023-08-22

**Authors:** Michael Frass, Gisela Etter-Kalberer, Michael Keusgen, Michaela Geiger, Rosemarie Brunnthaler-Tscherteu, Erfried Pichler, Bernhard Zauner, Menachem Oberbaum, Petra Weiermayer

**Affiliations:** 1 Institute for Homeopathic Research, Vienna, Austria; 2 WissHom: Scientific Society for Homeopathy, Wallstr. 48, Koethen, Germany; 3 Swiss Association of Homeopathic Physicians (SVHA), Richterswil/Zuerich, Switzerland; 4 Faculty of Pharmacy, Institute of Pharmaceutical Chemistry, Philipps-University Marburg, Marburg, Germany; 5 German Association of Homeopathic Doctors, Berlin, Germany; 6 Austrian Society for Homeopathic Medicine, Vienna, Austria; 7 Medical Society for Classical Homeopathy, Gmunden, Austria; 8 The Center for Integrative Complementary Medicine, Shaare Zedek Medical Center, Jerusalem, Israel

We read with great interest the work of Borkens et al. [1]. Contrary to the authors statement of “prescientific era”, the effectiveness of homeopathy has been shown and described in the literature [2, 3]. To start with basic research, in 70% of the 200 or so experiments studied, differences between homeopathic medicinal products (HMPs) and controls were observed according to systematic reviews of physicochemical test procedures. In the subgroup of high-quality experiments, 80% of the experiments showed differences between HMPs and controls. For 10 test procedures, 2–9 replicates each, which could be replicated 100% positively, were identified [4]. The study by Ücker et al. in 2022 provided further scientific evidence that HMPs have specific biological effects that are not due to a placebo effect. At the same time, this study confirmed the results of an earlier study [5]. In 2018, the systematic review of research on plant-based bioassays was updated. The authors identified 192 publications with 202 experimental studies. In the subgroup of experiments with adequate quality and appropriate controls for specific effects of HMPs, 95% of the studies showed significant differences compared with controls [6].

With respect to clinical research, there is a sufficient number of randomized, placebo-controlled trials in homeopathy. In all but one of the six large meta-analyses, effects of homeopathy beyond placebo were found [2]. Already immediately after the publication of the article by Shang et al. [7], it was shown by scientists of the Medical University of Vienna that this paper has undeniably serious scientific flaws [8]. While the authors classified homeopathy as prescientific, they obviously do not take into account the fact that more than 9 of 10 conventional therapies examined using Cochrane reviews are not based on high-quality evidence [9]. Contrary to the authors’ statement there is no risk that patients abandon effective medical treatment or prevention measures in favor of homeopathy as the treating physicians are trained in both conventional and homeopathic medicine and also have clinical experience, which according to the specifications of the first describers David Sackett et al., is one of the three pillars of evidence-based medicine in addition to patient preference and external clinical evidence [10]. Effectiveness, safety of and basic research in homeopathic medicine is substantially described in the literature and a placebo effect can be excluded.
